# An MMF3 Criterion Based Multi-Scale Strategy for the Failure Analysis of Plain-Woven Fabric Composites and Its Validation in the Open-Hole Compression Tests

**DOI:** 10.3390/ma14164393

**Published:** 2021-08-05

**Authors:** Rui Zhou, Weicheng Gao, Wei Liu

**Affiliations:** Department of Astronautics Science and Mechanics, Harbin Institute of Technology, Harbin 150001, China; zhourui3805@buaa.edu.cn (R.Z.); weiliu@hit.edu.cn (W.L.)

**Keywords:** multi-scale strategy, plain-woven fabric composites, finite-element method, representative unit cell, experimental validation

## Abstract

A modified micromechanics failure criterion (MMF3) based multi-scale analysis strategy was proposed in this article to analyze the failure behaviors of the plain-woven fabric composites. The finite-element (FE) representative unit cell (RUC) models of different scales were first established, and the RUC based stress transformation methods were developed. The micro-scale strengths of the constituents in the unidirectional laminate were achieved based on the tested macro-scale strengths. Under the micro-scale strength invariance hypothesis, the meso-scale strengths of the fiber tows from the plain-woven fabric composites were back-calculated first and were then validated and corrected with the assistance of tested strengths of the fabric laminates. With the micro-scale RUC and the calculated meso-scale strengths of the fiber tows, the micro-scale strengths of the constituents suitable for the plain-woven fabric composites were determined. The multi-scale analysis procedure for the plain-woven fabric composites was then established in providing a more direct failure observation at the constituent level. Open-hole compression specimens were tested according to the ASTM standard D6484, and the failure of the open-hole fabric laminate was simulated with the proposed multi-scale strategy. The numerical predictions were in good agreement with the experimental results, and the feasibility of the multi-scale strategy was validated.

## 1. Introduction

With high ratios of strength & stiffness to weight and outstanding corrosion resistance, fiber-reinforced composite materials are being widely used in modern air-plane structures. Plain-woven fabric composites are becoming increasingly important with the advantages of lower production costs and higher manufacturing efficiency. However, factors such as the architecture of fiber tows and the material properties of fiber and matrix constituents have considerable effects on the failure behavior and damage process of plain-woven fabric composite structures, making their damage analysis more complicated.

In order to understand the damage mechanisms and improve the design level, researches have been widely carried out on the damage process and failure behaviors of plain-woven fabric composite structures. An analytical model was presented by Donadon et al. [1] in predicting the elastic behavior of plain weave fabric composites with different materials and undulations in the warp and weft directions and good correlations were achieved between predictions and experimental results. Mesh generating is a focused issue in the modeling of woven fabric composites. Doitrand et al. [2] studied the applicability of voxel meshes in modeling the mechanical behaviors of plain-woven composites at the mesoscale. Grail et al. [3] presented a method in generating smooth FE meshes for the RUC of textile composites. The presented method allowed for efficient generation of consistent and conformal FE meshes with deformed, compacted, and nested textile reinforcements. Goyal et al. [4] studied the mechanical properties of 2 × 2 biaxial braided composites and the effects of parameters such as braid angle, waviness ratio, and material properties on the effective engineering properties of the 2 × 2 braids were investigated. Page et al. [5] developed the two-dimensional plane strain FE models in simulating the plane woven fabric composites. The effects of relative layer shift and laminate thickness on the stiffness properties and matrix cracking behavior were analyzed.

With the aim to have a broader ken in the investigations, multi-scale methods have been widely used in the study of woven composites. Matveev et al. [6] studied the effects of fiber strength variability on the mechanical properties of textile composites with multi-scale analytical methods. Obert et al. [7] constructed the micro-meso relations in enhancing the meso-scale damage model for 2D woven composites. Zhou et al. [8] studied the damage and failure behavior of 2D plain woven composites with a two-step multi-scale progressive damage analysis. With a formal-unified 3D Hashin-type criterion, the simulated results under on-axis uniaxial tension and compression showed good agreements with experimental results and the failure characteristics under different loading conditions were discussed. Wehrkamp-Richter et al. [9] proposed a simulation framework in accurately predicting the mechanical response of triaxial braided composites based on meso-scale FE models. The numerical predictions validated the framework and underlined its potential for further damage modeling.

Various experimental methods have also been utilized in the study of woven composites. With the technique of X-ray Micro Tomography, Naouar et al. [10] presented a direct method in determining meso-scale FE models. Carvalho et al. [11] investigated the compressive behavior of orthogonal 2D woven fabric composites with experimental methods and the damage initiation and propagation were studied respectively in detail.

With the advantage in explaining the failure mechanisms at the constituent level, micromechanics-based failure criteria are becoming increasingly appealing for researchers in modeling the failure behaviors of composite structures. By combining macro-scale FE models with RUCs of meso and micro scales, the multi-scale analysis method takes advantages of both the efficiency of the macro analysis and the accuracy of the micromechanics-based failure criteria. Goose et al. [12] proposed the Strain Invariant Failure Theory (SIFT) in predicting the onset of composites’ damage. In order to obtain the critical strain invariant values, strain enhancement factors are needed in the required linear finite element analysis [13]. Tran et al. [14] proposed a micromechanics-based modeling approach using the SIFT. Without the need for the strain enhancement factors, failure initiation sights could be identified directly to obtain the critical strain invariant values, making the multi-scale failure analysis procedure simpler. Mayes et al. [15] proposed the micromechanics-based Multi-continuum Theory (MCT) which associated a numerical algorithm for extracting the stress and strain fields of the composites’ constituents during a routine FE analysis. With the comparison between the MCT-based simulation results and the experimental results, the effectiveness of the MCT based method was generally validated [16]. Li et al. [17] proposed a stress-based multi-scale failure criterion with failure mechanisms concluded from experimental observations. Open-hole tension specimens were tested and the results were compared with numerical predictions in validating the proposed criterion. Ha et al. [18,19] proposed the micromechanics of failure (MMF) criterion in predicting the constituent failure of composite structures. The failure of fiber was determined by a maximum stress criterion and the failure of the matrix was determined by a revised von Mises criterion. In their further investigation [20] based on the MMF theory, micromechanical approaches were employed in investigating the effects of fiber arrangement on the mechanical behaviors of composite unidirectional laminates. Sun et al. [21] studied the progressive failure of open-hole composite laminates with modified MMF criterion and non-iterative element-failure method. The comparison between the numerical and experimental results demonstrated the efficiency and validity of the method. With a high-efficient MMF-based failure analysis model, Lou et al. [22] analyzed the fiber and matrix failure as well as interface debonding of macro composite structures under complex stress conditions. Considering the fiber-matrix interface in the micro-scale RUC, the failure analysis of this model showed high accuracy in the failure prediction. The multi-scale approaches combined with the MMF criterion have also been applied in predicting the damage initiation and propagation of woven fabric composite structures [23,24,25,26], indicating the huge potential of the MMF-based methods. In order to overcome the drawbacks of the MMF criterion in predicting matrix failure under longitudinal tension and shear strength, Sihn [27] proposed the modified micromechanics of failure (MMF3) criterion in improving the prediction accuracy. Liu et al. [28] proposed an MMF3-based multi-scale strategy with the thermal residual strength taken into account. FE models of composite laminates under off-axial loading were analyzed and the numerical predictions were validated by the experimental results.

Considering its advantage in prediction accuracy, the MMF3 criterion is utilized in this article in developing a multi-scale strategy used for the damage analysis of plain-woven fabric composites of T300/Cycom970. Micro-scale and meso-scale RUCs of plain-woven composites are established, and methods are developed in stress transformation. With the assistance of the RUCs and the macro-scale experimental data of strengths, the necessary strengths for multi-scale analysis are achieved and the MMF3-based multi-scale strategy for the damage analysis of plain-woven fabric composites is then developed. Specimens of open-hole composite laminates made of T300/Cycom970 plain woven fabrics are tested under compression. The corresponding multi-scale FE analysis is simultaneously implemented, and the numerical damage predictions are validated by the experimental results, indicating the effectiveness of the strategy.

## 2. Stress Transformation and the MMF3 Criterion

### 2.1. Stress Transformation

With the aim to determine the damage initiation at the micro level, stresses calculated from the macro-scale FE models need to be transferred to micro-scale stresses at the fiber and matrix constituents first.

#### 2.1.1. Stress Transformation for Unidirectional Tapes and Fiber Tows

For the composites of unidirectional tapes and fiber tows, the micro-scale stress vector **σ** at the reference point of the constituent region can be obtained directly from the macro-scale stress vector $\bar{\sigma }$ with the assistance of stress amplification factors (SAFs) [18]:

(1) $$\sigma =M\cdot \bar{\sigma }+A\cdot \Delta T$$

In Equation (1), **M** (6 × 6) stands for the matrix of mechanical SAFs and **A** (6 × 1) stands for the matrix of thermal SAFs.

The complete form of Equation (1) can be written as the following form of (2) [28].

(2) $$\left\{\begin{array}{c} \sigma_{11}\\ \sigma_{22}\\ \sigma_{33}\\ \tau_{12}\\ \tau_{13}\\ \tau_{23} \end{array}\right\}=\left[\begin{array}{cccccc} M_{11} & M_{12} & M_{13} & 0 & 0 & M_{16}\\ M_{21} & M_{22} & M_{23} & 0 & 0 & M_{26}\\ M_{31} & M_{32} & M_{33} & 0 & 0 & M_{36}\\ 0 & 0 & 0 & M_{44} & M_{45} & 0\\ 0 & 0 & 0 & M_{54} & M_{55} & 0\\ M_{61} & M_{62} & M_{63} & 0 & 0 & M_{66} \end{array}\right]\left\{\begin{array}{c} {\bar{\sigma }}_{11}\\ {\bar{\sigma }}_{22}\\ {\bar{\sigma }}_{33}\\ {\bar{\tau }}_{12}\\ {\bar{\tau }}_{13}\\ {\bar{\tau }}_{23} \end{array}\right\}+\left[\begin{array}{c} A_{11}\\ A_{21}\\ A_{31}\\ 0\\ 0\\ A_{61} \end{array}\right]\Delta T$$

**Figure 1:**
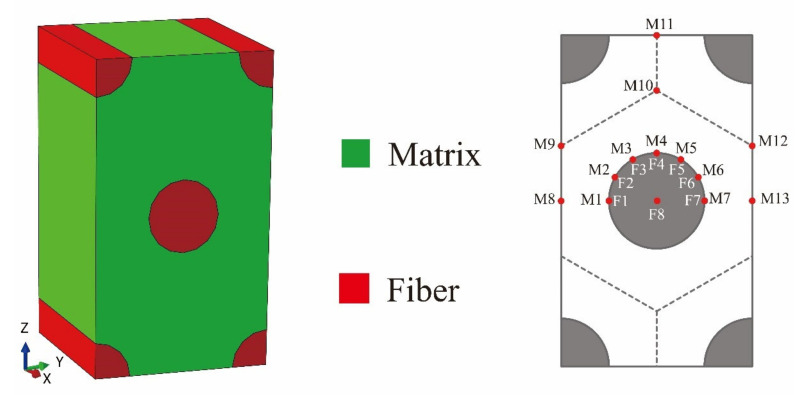
The hexagonal micro-scale RUC and reference points of the constituents.

The proper FE model of RUC is the necessary tool in the transformation of the stresses. The hexagonal assumption has been proved to be accurate enough in modeling the real fiber array of composites [29] and the hexagonal micro-scale RUC has a wilder modeling range of the composites’ fiber volume fraction. Considering the above advantages, the hexagonal micro-scale RUC is used in this paper as shown in Figure 1. 

The reference points of the constituents which can cover the dangerous situations during the calculation are also illustrated in Figure 1. Points of F1–F8 are chosen in the fiber region and points of M1–M13 are chosen in the matrix region.

**Figure 2:**
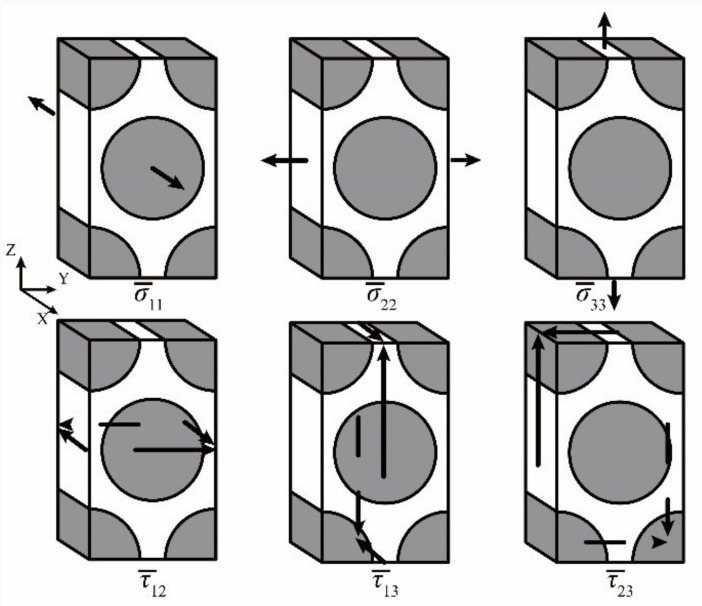
Macro-scale stresses applied on the micro-scale RUC in obtaining stress amplification factors (SAFs) of **M** matrix.

For each reference point, the mechanical SAFs in the matrix of M can be calculated with the macro-scale stresses applied on the micro-scale RUC, as shown in Figure 2. The SAFs in the matrix of A can be obtained by achieving the micro-scale stresses with unit temperature increment applied on the constrained micro-scale RUC.

#### 2.1.2. Stress Transformation for Plain-Woven Fabric Composites

**Figure 3:**
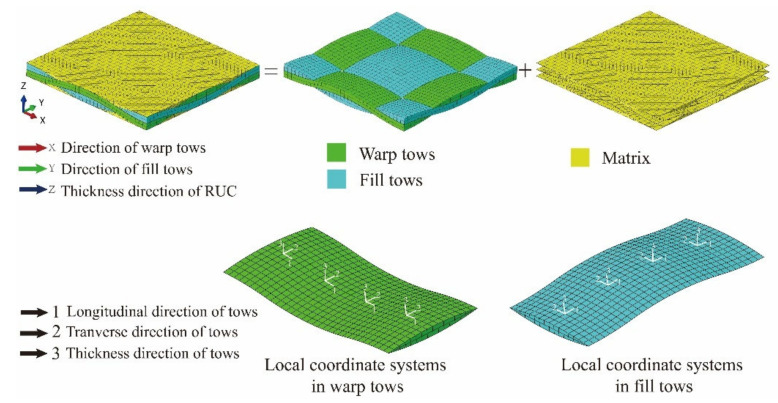
The meso-scale RUC of the plain-woven fabric composites.

For the plain-woven fabric composites, the meso-scale RUC is divided into regions of fiber tows and pure matrix, as is shown in Figure 3. In order to achieve the micro-scale stresses of the constituents and evaluate the matrix damage, the meso-scale stresses in the region of fiber tows (${\bar{\sigma }}^{t}$) and the meso-scale stresses in the pure matrix region (${\bar{\sigma }}^{m}$) need to be transferred from the macro-scale stresses ($\bar{\sigma }$) with the assistance of the meso-scale RUC first:

(3) $${\bar{\sigma }}^{t}=M^{t}\cdot \bar{\sigma }+A^{t}\cdot \Delta T$$

(4) $${\bar{\sigma }}^{m}=M^{m}\cdot \bar{\sigma }+A^{m}\cdot \Delta T$$

**Figure 4:**
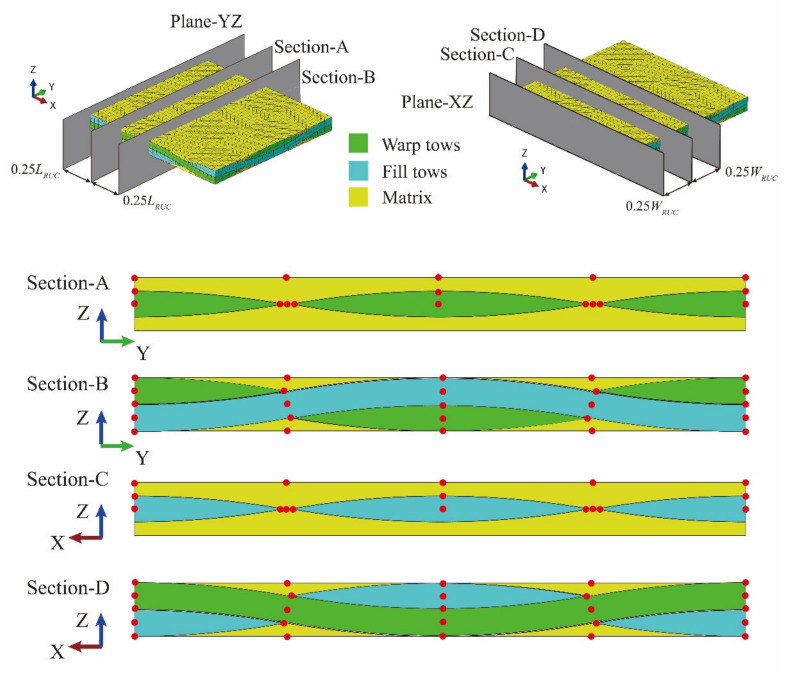
Reference points in the meso-scale RUC.

With the macro-scale stresses similar to Figure 2 and the unit temperature increment applied on the meso-scale RUC, the corresponding meso-scale stresses at the reference points in Figure 4 can be obtained. The SAFs of **M***^t^* and **A***^t^* in the region of fiber tows and the SAFs of **M***^m^* and **A***^m^* in the region of the pure matrix could then be calculated, indicating the establishment of the transformation methods between the macro-scale stresses and the meso-scale stresses at the reference points in Figure 4.

For ${\bar{\sigma }}^{m}$ in the pure matrix region of the meso-scale RUC, the meso-scale stresses remain the same in the micro-scale level and the stresses could be used directly in the determination of the micro-scale damage. For ${\bar{\sigma }}^{t}$ in the region of fiber tows, the meso-scale stresses could be further transferred to the micro-scale stresses (**σ**) of the fiber and matrix constituents with the method introduced in Section 2.1.1.

### 2.2. The MMF3 Criterion

Comparing to the MMF criterion [18], an independent micro-scale shear strength of matrix Sm is defined in improving the accuracy of the matrix damage determination in the MMF3 criterion [27]. Besides, considering that the fibers mainly bear the longitudinal load, the micro-scale stress σ_11 is set to have no effects on the damage initiation of the matrix in the MMF3 criterion in presenting a more rational prediction of longitudinal tensile strength.

In the MMF3 criterion, the failures of the fiber and matrix constituents are defined at the micro-scale level as shown in Table 1.

The micro-scale stresses transferred from the macro-scale stresses and the meso-scale stresses are utilized with the micro-scale strengths in determining the micro-scale failures. *T_f_*, *C_f_*, *T_m_*_,_ and *C_m_* represent the micro-scale tensile and compressive strengths of fiber and matrix respectively and *S_m_* represents the micro-scale shear strength of the matrix. $\sigma_{I}$, $\sigma_{II}$ and $\sigma_{III}$ are the compositions of the micro-scale stresses of $\sigma_{22}$, $\sigma_{33}$ and $\tau_{23}$ as follows:

(5) $$\sigma_{I}=\sigma_{22}+\sigma_{33}$$

(6) $$\sigma_{II}={\left(\sigma_{22}+\sigma_{33}\right)}^{2}$$

(7) $$\sigma_{III}=\sigma_{22}\sigma_{33}-\left(\tau_{12}{}^{2}+\tau_{13}{}^{2}+\tau_{23}{}^{2}\right)$$

## 3. Determination of the Strengths and Establishment of the Multi-Scale Strategy

In the multi-scale damage analysis of the plain-woven fabric composites with the MMF3 criterion, it is essential to determine the micro-scale strengths after the establishment of the stress transformation methods. The plain-woven fabric composite of T300/Cycom970 is chosen as the study object in this paper. The basic strength data have been obtained from the tests on T300/Cycom970 laminates made of unidirectional tapes and plain-woven fabrics first. With further calculations and corrections based on the experimental data and the FE models of RUCs, the micro-scale strengths suitable for the plain-woven fabric composite (T300/Cycom970) are then determined.

### 3.1. Micro-Scale Strengths of the Unidirectional Laminates

With the architecture characteristics of the plain-woven fabric composites, there are challenges in obtaining the micro-scale strengths directly from experimental strength data of fabric laminates. Based on the experimental macro-scale strengths of the T300/Cycom970 unidirectional laminates, the micro-scale strengths of the constituents are first achieved with the ordinary method.

**Figure 5:**
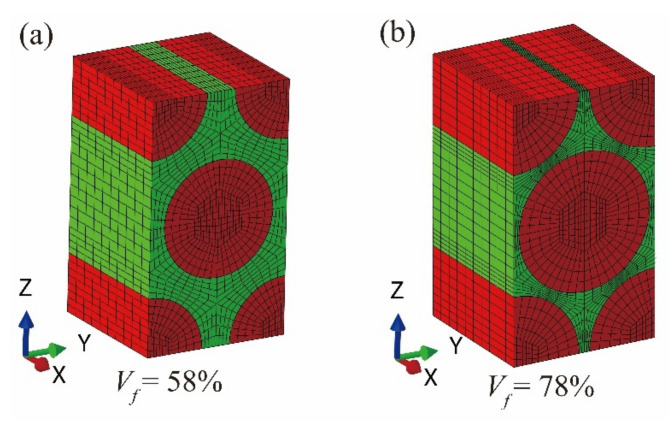
Hexagonal micro-scale RUCs with different values of fiber volume fraction: (**a**) RUC with fiber volume fraction of 58%; (**b**) RUC with fiber volume fraction of 78%.

The micro-scale RUC of the T300/Cycom970 unidirectional laminate is established as shown in Figure 5a with the mechanical properties of the fiber and the matrix [30] presented in Table 2. The value of the tested fiber volume fraction of the unidirectional laminate *V_f_* is 0.58. 10,240 elements of C3D8R are utilized in the micro-scale RUC. The subscripts *f* and *m* of the parameters listed in Table 2 represent the constituents of fiber and matrix respectively.

**Figure 6:**
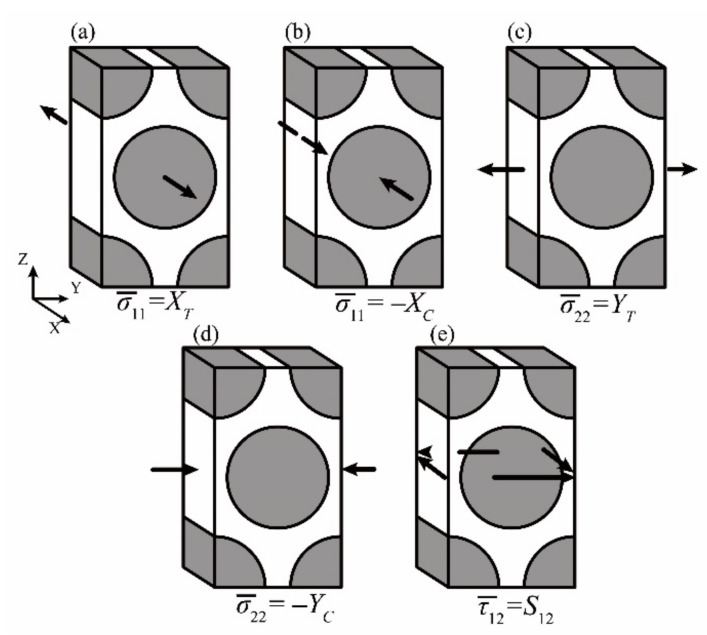
Micro-scale RUC under different loading conditions of macro-scale strengths: (**a**) RUC under *X_T_*; (**b**) RUC under—*X_C_*; (**c**) RUC under *Y_T_*; (**d**) RUC under—*Y_C_*; (**e**) RUC under *S*_12_.

The tested macro-scale strengths of the T300/Cycom970 unidirectional laminates are listed in Table 3 and are applied on the boundaries of the micro-scale RUC as shown in Figure 6. Under each of the loading conditions, the corresponding micro-scale stresses at the reference points in Figure 1 could be calculated with the stress transformation method introduced in Section 2.1.1. With the calculated micro-scale stresses, micro-scale strengths of the T300/Cycom970 unidirectional laminates can be obtained.

#### 3.1.1. Determination of *T_f_* and *C_f_*

In Figure 6a, with the macro-scale longitudinal tensile strength of *X_T_* applied on the micro-scale RUC, the micro-scale failure of fiber initiates corresponding to the macro-scale longitudinal tensile failure of the unidirectional laminates. Considering the MMF3 criterion, the maximum micro-scale stress of $\sigma_{11}$ reaches the micro-scale tensile strength of fiber *T_f_* in this condition:

(8) $$\sigma_{11}{}^{\left(i\right)}=M_{11}{}^{\left(i\right)}\cdot X_{T}+A_{11}{}^{\left(i\right)}\cdot \Delta T$$

(9) $$T_{f}=max(M_{11}{}^{\left(i\right)}\cdot X_{T}+A_{11}{}^{\left(i\right)}\cdot \Delta T)$$

where *i* = 1, 2 … 8 denotes the reference points in the fiber region of the micro-scale RUC and ΔT denotes the difference between the curing temperature of the T300/Cycom970 prepregs and the room temperature.

Similarly, *C_f_* can be obtained with the calculated micro-scale stresses of the reference points in the matrix region under the macro-scale longitudinal compressive strength of −*X_C_* as shown in Figure 6b:

(10) $$\sigma_{11}{}^{\left(i\right)}=-M_{11}{}^{\left(i\right)}\cdot X_{C}+A_{11}{}^{\left(i\right)}\cdot \Delta T$$

(11) $$-C_{f}=min(-M_{11}{}^{\left(i\right)}\cdot X_{C}+A_{11}{}^{\left(i\right)}\cdot \Delta T)$$

#### 3.1.2. Determination of *S_m_*

In Figure 6c, with the macro-scale shear strength of *S*_12_ applied on the micro-scale RUC, the micro-scale failure of matrix initiates corresponding to the macro-scale shear failure of the unidirectional laminates. Under this loading condition, the micro-scale stresses of the reference point *i* = 1, 2 … in the matrix region of the micro-scale RUC include the following components:

(12) $$\tau_{12}{}^{\left(i\right)}=M_{44}{}^{\left(i\right)}\cdot S_{12}$$

(13) $$\tau_{13}{}^{\left(i\right)}=M_{54}{}^{\left(i\right)}\cdot S_{12}$$

According to the MMF3 criterion, the micro-scale matrix failure initiates when the following condition is satisfied after going through all the reference points in the matrix region:

(14) $$max\left\{\frac{1}{S_{m}{}^{2}}\cdot \left[{\left(\tau_{12}{}^{\left(i\right)}\right)}^{2}+{\left(\tau_{13}{}^{\left(i\right)}\right)}^{2}\right]\right\}=1$$

With the consideration of Equations (12) and (13), the micro-scale shear strength of matrix is then determined:

(15) $$S_{m}=max(\sqrt{{\left(M_{44}{}^{\left(i\right)}\cdot S_{12}\right)}^{2}+{\left(M_{54}{}^{\left(i\right)}\cdot S_{12}\right)}^{2}})$$

#### 3.1.3. Determination of *T_m_* and *C_m_*

In Figure 6d,e, with the macro-scale transverse tensile and compressive strengths of *Y_T_* and −*Y_C_* applied on the micro-scale RUC, the micro-scale failures of the matrix initiate corresponding to the macro-scale transverse tensile and compressive failures of the unidirectional laminates. With Equation (2), the macro-scale stresses are transferred to micro-scale stresses at the reference points:

(16) $$\sigma_{22}{}^{T\left(i\right)}=M_{22}{}^{\left(i\right)}\cdot Y_{T}+A_{21}{}^{\left(i\right)}\cdot \Delta T,\sigma_{22}{}^{C\left(i\right)}=-M_{22}{}^{\left(i\right)}\cdot Y_{C}+A_{21}{}^{\left(i\right)}\cdot \Delta T$$

(17) $$\sigma_{33}{}^{T\left(i\right)}=M_{32}{}^{\left(i\right)}\cdot Y_{T}+A_{31}{}^{\left(i\right)}\cdot \Delta T,\sigma_{33}{}^{C\left(i\right)}=-M_{32}{}^{\left(i\right)}\cdot Y_{C}+A_{31}{}^{\left(i\right)}\cdot \Delta T$$

(18) $$\tau_{23}{}^{T\left(i\right)}=M_{62}{}^{\left(i\right)}\cdot Y_{T}+A_{61}{}^{\left(i\right)}\cdot \Delta T,\tau_{23}{}^{C\left(i\right)}=-M_{62}{}^{\left(i\right)}\cdot Y_{C}+A_{61}{}^{\left(i\right)}\cdot \Delta T$$

In the above equations, the superscripts *T* and *C* represent the transverse tensile and compressive loading conditions corresponding to Figure 6d,e, respectively.

Referring to Equations (5)–(7), the micro-scale stress compositions corresponding to the above transverse loading conditions are expressed as:

(19) $$\sigma_{I}{}^{T\left(i\right)}=\sigma_{22}{}^{T\left(i\right)}+\sigma_{33}{}^{T\left(i\right)},\sigma_{I}{}^{C\left(i\right)}=\sigma_{22}{}^{C\left(i\right)}+\sigma_{33}{}^{C\left(i\right)}$$

(20) $$\sigma_{II}{}^{T\left(i\right)}={\left(\sigma_{22}{}^{T\left(i\right)}+\sigma_{33}{}^{T\left(i\right)}\right)}^{2},\sigma_{II}{}^{C\left(i\right)}={\left(\sigma_{22}{}^{C\left(i\right)}+\sigma_{33}{}^{C\left(i\right)}\right)}^{2}$$

(21) $$\sigma_{III}{}^{T\left(i\right)}=\sigma_{22}{}^{T\left(i\right)}\cdot \sigma_{33}{}^{T\left(i\right)}-{\left(\tau_{23}{}^{T\left(i\right)}\right)}^{2},\sigma_{III}{}^{C\left(i\right)}=\sigma_{22}{}^{C\left(i\right)}\cdot \sigma_{33}{}^{C\left(i\right)}-{\left(\tau_{23}{}^{C\left(i\right)}\right)}^{2}$$

In satisfying the micro-scale matrix failure criterion in Table 1, with $\beta_{m}=C_{m}/T_{m}$ imported as a temporary variable, *T_m_* and *C_m_* are expressed as:

(22) $$T_{m}=\frac{\left(\beta_{m}-1\right)\sigma_{I}+\sqrt{{\left(\beta_{m}-1\right)}^{2}\sigma_{I}{}^{2}+4\left(\sigma_{III}/S_{m}{}^{2}+1\right)\beta_{m}\sigma_{II}}}{2\left(\sigma_{III}/S_{m}{}^{2}+1\right)\beta_{m}}$$

(23) $$C_{m}=\frac{\left(\beta_{m}-1\right)\sigma_{I}+\sqrt{{\left(\beta_{m}-1\right)}^{2}\sigma_{I}{}^{2}+4\left(\sigma_{III}/S_{m}{}^{2}+1\right)\beta_{m}\sigma_{II}}}{2\left(\sigma_{III}/S_{m}{}^{2}+1\right)}$$

Under the transverse tensile loading condition shown in Figure 6c, after going through all the reference points in the matrix region with $\beta_{m}=\beta_{m}{}^{0}$, the corresponding micro-scale tensile strength of matrix could be calculated and expressed as:

(24) $$T_{m}{}^{0}=max[\frac{\left(\beta_{m}{}^{0}-1\right)\sigma_{I}{}^{T\left(i\right)}+\sqrt{{\left(\beta_{m}{}^{0}-1\right)}^{2}{\left(\sigma_{I}{}^{T\left(i\right)}\right)}^{2}+4\left(\sigma_{III}{}^{T\left(i\right)}/S_{m}{}^{2}+1\right)\beta_{m}{}^{0}\sigma_{II}{}^{T\left(i\right)}}}{2\left(\sigma_{III}{}^{T\left(i\right)}/S_{m}{}^{2}+1\right)\beta_{m}{}^{0}}]$$

Similarly, with $\beta_{m}=\beta_{m}{}^{0}$ under the loading condition in Figure 6d, the corresponding micro-scale compressive strength of matrix can be calculated and expressed as:

(25) $$C_{m}{}^{0}=max[\frac{\left(\beta_{m}{}^{0}-1\right)\sigma_{I}{}^{C\left(i\right)}+\sqrt{{\left(\beta_{m}{}^{0}-1\right)}^{2}{\left(\sigma_{I}{}^{C\left(i\right)}\right)}^{2}+4\left(\sigma_{III}{}^{C\left(i\right)}/S_{m}{}^{2}+1\right)\beta_{m}{}^{0}\sigma_{II}{}^{C\left(i\right)}}}{2\left(\sigma_{III}{}^{C\left(i\right)}/S_{m}{}^{2}+1\right)}]$$

Slightly difference should exist between the reasonable value of $\beta_{m}{}^{0}$ and the ratio of $\beta_{m}{}^{1}=C_{m}{}^{0}/T_{m}{}^{0}$. In this paper, the tolerance of the difference is set as 0.001 to guarantee the accuracy in the determinations of $T_{m}=T_{m}{}^{0}$ and $C_{m}=C_{m}{}^{0}$.

**Figure 7:**
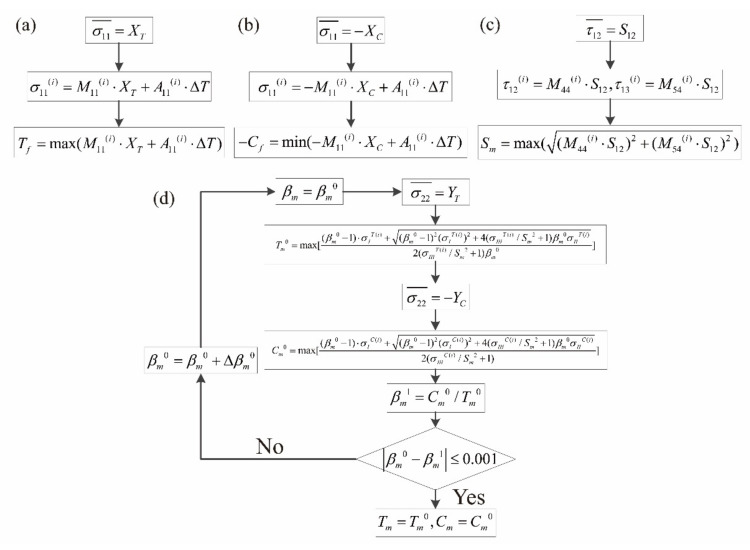
Procedures in determining the micro-scale strengths with macro-scale strengths of unidirectional laminates: (**a**) procedure in determining *T_f_*; (**b**) procedure in determining *C_f_*; (**c**) procedure in determining *S_m_*; (**d**) procedure in determining *T_m_* and *C_m_*.

The procedures in determining the micro-scale strengths of the constituents with tested macro-scale strengths of the unidirectional composite laminates are illustrated in Figure 7. With the macro-scale strengths shown in Table 3, the micro-scale strengths of the T300/Cycom970 unidirectional laminates are determined and presented in Table 4.

### 3.2. Strengths of the Plain-Woven Fabric Composites

In order to calculate the micro-scale strengths suitable for the plain-woven fabric composites with the MMF3 based method similar to Figure 7, it is necessary to obtain the meso-scale strengths of fiber tows in the fabrics.

#### 3.2.1. Meso-Scale Strengths of Fiber Tows Calculated under the Micro-Scale Strength Invariant Hypothesis

The tested mechanical properties of the T300/Cycom970 plain-woven composite laminates are shown in Table 5 with the subscript *F* representing the parameters of the fabrics. Under the micro-scale strength invariant hypothesis that the micro-scale strengths of the constituents do not change with the variation of the fiber volume fraction, the meso-scale strengths of fiber tows in the T300/Cycom970 fabrics are first back-calculated with the micro-scale strengths listed in Table 4.

**Figure 8:**
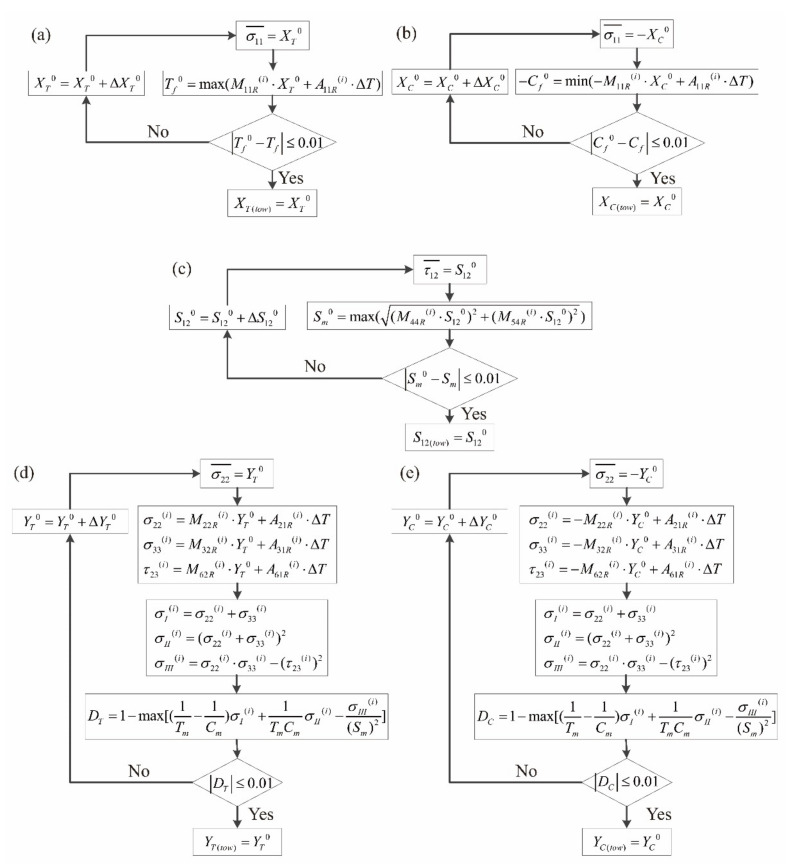
Procedures in determining the meso-scale strengths of fiber tows under the micro-scale strength invariant hypothesis: (**a**) procedure in determining *X_T(tow)_*; (**b**) procedure in determining *X_C(tow)_*; (**c**) procedure in determining *S*_12*(tow)*_; (**d**) procedure in determining *Y_T(tow)_*; (**e**) procedure in determining *Y_C(tow)_*.

As the fiber volume fraction changes to *V_f_* = 0.78 in the fiber tows, the corresponding micro-scale RUC has been reestablished as shown in Figure 5b, with the constituents’ mechanical properties and the quantity of the elements unchanged. The matrixes of SAFs at the different reference points i have been recalculated as **M**_R_^(i)^ and **A**_R_^(i)^. In Figure 8, the procedures in calculating the meso-scale strengths of fiber tows with the micro-scale strengths of the constituents are presented. Figure 8a is taken as an example in calculating the meso-scale longitudinal tensile strength of fiber tows *X_T(tow)_*. Considering Equation (9) and import the macro-scale longitudinal tensile strength of fiber tows with an initial value *X_T_*^0^, the corresponding micro-scale tensile strength of the fiber is obtained:

(26) $$T_{f}{}^{0}=max(M_{11}{{}_{R}}^{\left(i\right)}\cdot X_{T}{}^{0}+A_{11}{{}_{R}}^{\left(i\right)}\cdot \Delta T)$$

Under the micro-scale strength invariant hypothesis, $T_{f}{}^{0}$ in Equation (24) calculated with the proper *X_T_*^0^ equals to the *T_f_* value listed in Table 4. The absolute value of the difference between $T_{f}{}^{0}$ and *T_f_* will finally be controlled under the limit of 0.001 with the iteration method, and the corresponding value of *X_T_*^0^ is then determined as the meso-scale longitudinal tensile strength of fiber tows *X_T(tow)_*.

With similar methods illustrated in Figure 8b–e, the calculated meso-scale strengths of fiber tows based on the micro-scale strengths in Table 4 and the micro-scale RUC in Figure 5b are presented in Table 6.

#### 3.2.2. Validation and Correction on the Meso-Scale Strengths of Fiber Tows

**Figure 9:**
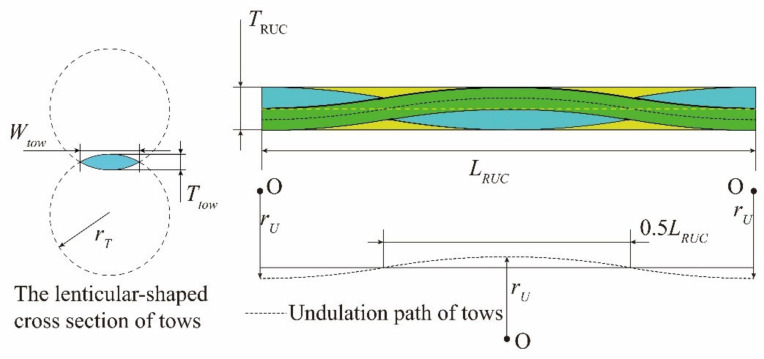
Geometric parameters in establishing the meso-scale RUC.

In validating and correcting the meso-scale strengths from Table 6, the meso-scale RUC of T300/Cycom970 fabrics has been established and calculations based on experimental strengths have been carried out. The fiber tows in the RUC are modeled with a lenticular-shaped cross-section [31] which sweeps along the arc-shaped undulation path [26]. The geometric parameters utilized in establishing the meso-scale RUC are illustrated in Figure 9.

In Figure 9, *W_tow_*, *T_tow_*, and *r_tow_* are controlling parameters that define the lenticular-shaped cross-section of the fiber tows. The arc-shaped undulation path of the fiber tows is controlled by the radius of *r_U_*. *L_RUC_*, *W_RUC_*_,_ and *T_RUC_* represent the length, width, and thickness of the meso-scale RUC, respectively, with *W_RUC_* equals to *L_RUC_* considering the plain-woven structure of the studied fabrics. Referring to the relevant literature [25,26,27], and the observation results with Micro CT, the values of the above parameters are determined in Table 7.

The pure matrix region of the meso-scale RUC is discretized with the tetrahedral elements of C3D4 in suiting its geometric complexity and guaranteeing the calculation accuracy. In discretizing the regular region of fiber tows, the hexahedral elements of C3D8R are utilized in enhancing the calculation efficiency with a smaller amount of meshes. 25,668 elements are finally used in the meso-scale RUC as presented in Figure 3, which led to both a computation-efficient and accuracy-guaranteed mesh size. 3840 elements are hexahedral elements of C3D8R which model the fiber tows and 21,828 elements are tetrahedral elements of C3D4 which models the pure matrix. For the region of fiber tows, the mechanical properties are calculated with the micro-scale RUC in Figure 5b using the properties of the constituents listed in Table 2. The values of the properties are presented in Table 8 and assigned to the elements of fiber tows with the consideration of the local coordinates as shown in Figure 3. For the region of the pure matrix in the meso-scale RUC, the mechanical properties are consistent with the properties of the matrix listed in Table 2. Using the platform of Intel Core i7-9750H CPU in validating and correcting the meso-scale strengths of the fiber tows, the single run time of the meso-scale RUC model is 16 s.

**Figure 10:**
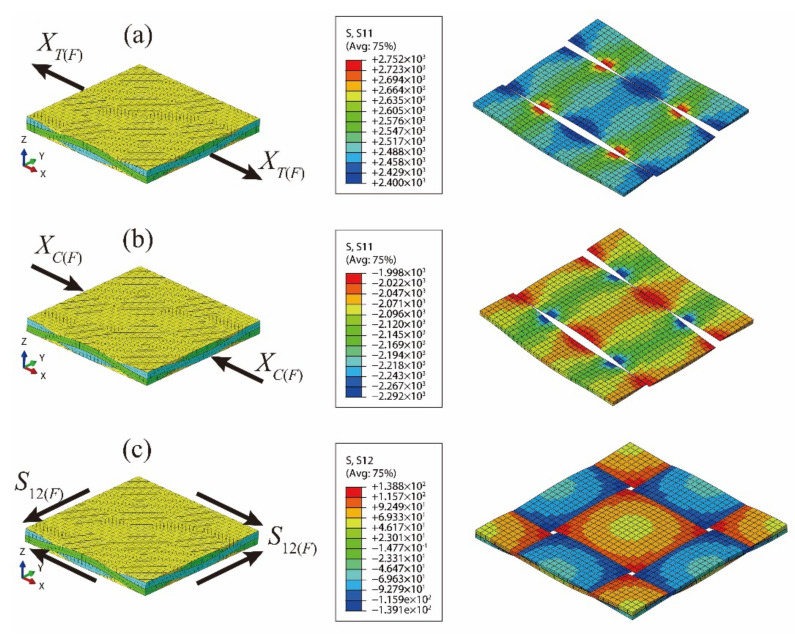
Numerical stresses of the fiber tows calculated with the meso-scale RUC under the macro-scale strengths of the fabric laminates: (**a**) numerical result of RUC under *X_T(F)_*; (**b**) numerical result of RUC under *X_C(F)_*; (**c**) numerical result of RUC under *S*_12*(F)*_.

Macro-scale strengths of the T300/Cycom970 fabric laminates (Table 5) have been applied to the established meso-scale RUC in order to calculate the corresponding stress results as the meso-scale strengths of the fiber tows. The loading conditions and the corresponding numerical results are presented in Figure 10. As the fill fiber tows of the meso-scale RUC mainly bear the transverse load under the loading conditions of *Y_T(F)_* and *Y_C(F)_*, the corresponding calculated stresses in the fill fiber tows are similar to results in the warp fiber tows of the meso-scale RUC under the longitudinal load of *X_T(F)_* and *X_C(F)_*. With the above characteristics, transverse strengths of the fiber tows (*Y_T(tow)_* and *Y_C(tow)_*) cannot be validated with the calculation method based on the meso-scale RUC and the macro-scale strengths of fabric laminates.

**Figure 11:**
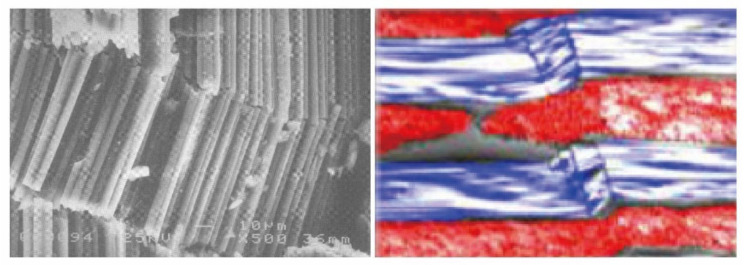
Typical failure modes of composite unidirectional laminates and fabric laminates [11,32].

The calculated meso-scale strengths with the above method are listed in Table 9 and compared with the results based on the micro-scale strength invariant hypothesis. The meso-scale strength of *X_C(tow)_* obtained from the micro-scale strength invariant hypothesis is smaller. Typical failure modes of composite unidirectional laminates [32] and fabric laminates [11] under axial compression are presented in Figure 11. As the fiber micro-buckling caused kink band stands the leading role in the compressive failures of the composites, the lateral support provided by the fill tows will enhance the longitudinal compressive strength of the fiber tows by postponing the initiation of the fiber micro-buckling and the subsequent kink band. The effect of lateral support provided by the fill tows is not considered in the micro-scale strength invariant hypothesis, leading to the smaller prediction of the longitudinal compressive strength of fiber tows *X_C(tow)_*. The other two meso-scale strength parameters are validated since the meso-scale strengths of *X_T(tow)_* and *S*_12*(tow)*_ obtained from the two methods are close to each other.

The corrected meso-scale strengths of the fiber tows in the studied T300/Cycom970 fabrics are listed in Table 10, along with the corresponding micro-scale strengths of the constituents suitable for the T300/Cycom970.

### 3.3. The Multi-Scale Strategy for the Failure Analysis of the Plain-Woven Fabrics

**Figure 12:**
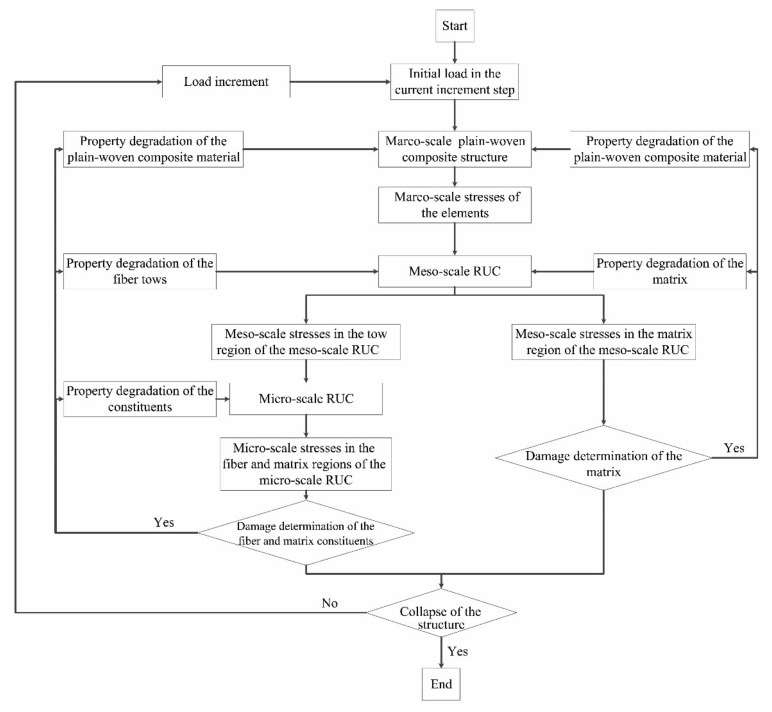
Flowchart of implementing the MMF3-based procedure in the multi-scale failure analysis of plain-woven fabric composites.

With the above methods of stress transformation and the essential micro-scale strengths of the constituents, the multiscale strategy for the failure analysis of the plain-woven fabric composites has been developed as illustrated in Figure 12.

At the beginning of each load increment, the stresses of the elements in the macro-scale FE models are calculated first and then transferred into the meso-scale stresses at the reference points of the meso-scale RUC with the method illustrated in Section 2.1.2. For the region of the pure matrix in the meso-scale RUC, since the meso-scale stresses are equal to the micro stresses, they are imported directly into the MMF3 criterion in determining the micro-scale failure of the matrix. For the region of the fiber tows in the meso-scale RUC, the meso-scale stresses are further transferred into micro-scale stresses at the constituents’ reference points with the method illustrated in Section 2.1.1 based on the micro-scale RUC shown in Figure 5b. The micro-scale stresses are then imported into the MMF3 criterion in determining the micro-scale failure of the constituents in the region of the fiber tows.

Once the micro-scale stresses satisfy the MMF3 criterion in Table 1, the damage initiates, and the properties of the corresponding constituent are degraded in implementing the progressive damage. The degradation methods [28] utilized in this paper are presented in Table 11. 

As the properties of the constituents degrade, the mechanical properties of the matrix and the fiber tows in the meso-scale RUC degrade correspondingly, along with the degradation of the macro-scale mechanical properties of the plain-woven fabric composites. In the following load increment, the recalculated SAFs based on the degraded RUC models are utilized in the transformation of the stresses and the MMF3 criterion is further utilized in the determination of damage evolution. The above procedures cycle until a sharp decrease of load occurs, which indicates the collapse of the specimen.

Compression experiments have been performed on the open-hole composite laminates made of T300/Cycom970 plain-woven fabrics. The experimental results are compared with the numerical results of the multi-scale finite element analysis in order to validate the above proposed methodology.

## 4. Experiments of Open-Hole Composite Laminates

### 4.1. Compression Experiments

**Figure 13:**
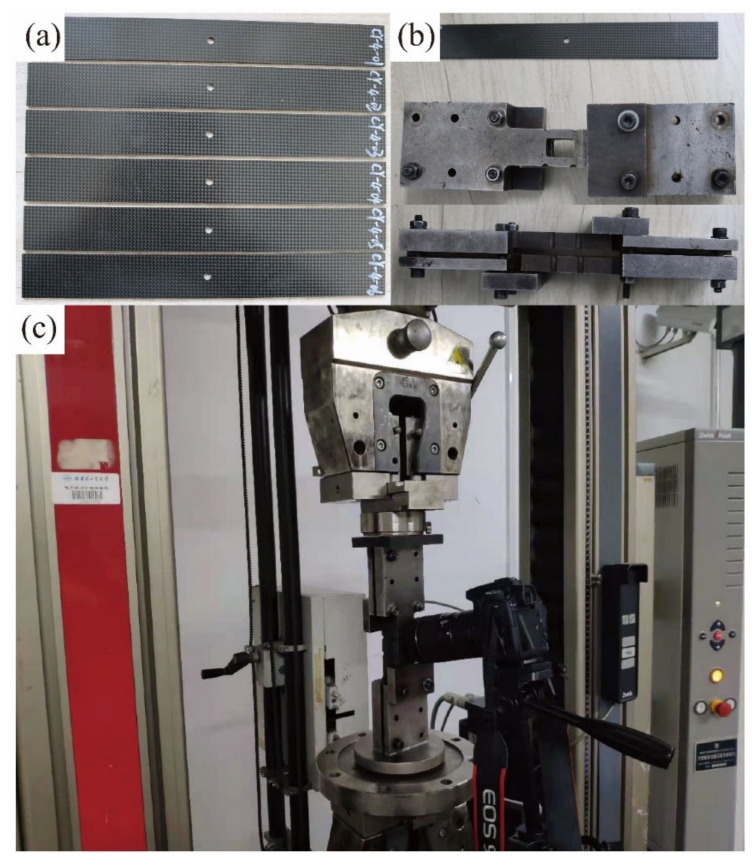
Specimens of the T300/Cycom970 fabric open-hole laminates and the loading system of the compression experiments: (**a**) the specimens of the open-hole laminates; (**b**) the fixture of the open-hole compression experiments; (**c**) the testing machine.

With the aim to validate the proposed multi-scale strategy, the manufacturing process of the open-hole laminates was intentionally controlled in enhancing the consistency of the specimens and reducing the uncertainty effects. The fabric prepregs used in manufacturing the open-hole laminates and the basic test specimen were from the same batch. The heat-press technological condition maintained consistency. All the open-hole laminates came from the same plate and were cut and drilled by the same staff under the same process. The specimens of the open-hole laminates with the stacking sequence of [45/0]_4s_ are shown in Figure 13a. The dimension of the specimens is 300 mm × 36 mm × 3.456 mm and the diameter of the central hole is 6 mm. In the experiments, the specimens were clamped with the fixture presented in Figure 13b and were tested under the ASTM Standard D6484 [33] based procedure. The compression experiments were conducted on an electronic universal testing machine with a maximum loading capacity of 100 kN, as shown in Figure 13c. Six specimens were tested in the experiments with the load-shortening curves automatically recorded by the testing machine. The progressive damage of the open-hole region of the laminates was simultaneously recorded using the method of macro photography.

### 4.2. Experimental Results

**Figure 14:**
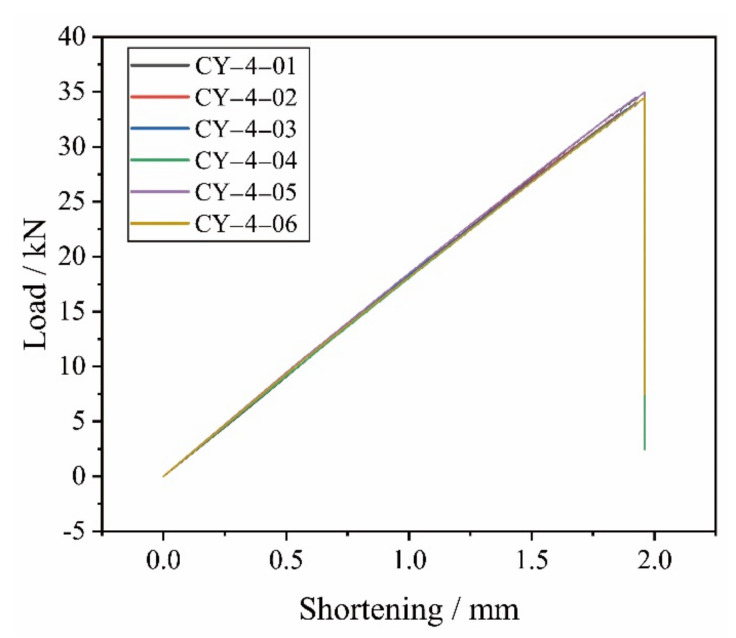
Experimental load-shortening curves of the specimens.

In Table 12, the experimental collapse loads of the specimens are listed, along with the average value of the collapse loads, the standard deviation, and the coefficient of dispersion. The effects of the above control methods are reflected by the consistency of the experimental results. With the small values of the standard deviation and the coefficient of dispersion, the experimental results present good repeatability, which can also be detected from the experimental load-shortening curves as shown in Figure 14. With the neglectable effects of uncertainty, the specimens behaved well in the aspect of validation. The experimental load-shortening curve of the CY-4-01 specimen corresponds well with the curves of the other specimens and the collapse load value of the CY-4-01 specimen is close to the average collapse value of 34.7 kN. In order to simplify the following analysis, the results of the CY-4-01 specimen are chosen as the representative experimental results in the following discussion.

**Figure 15:**
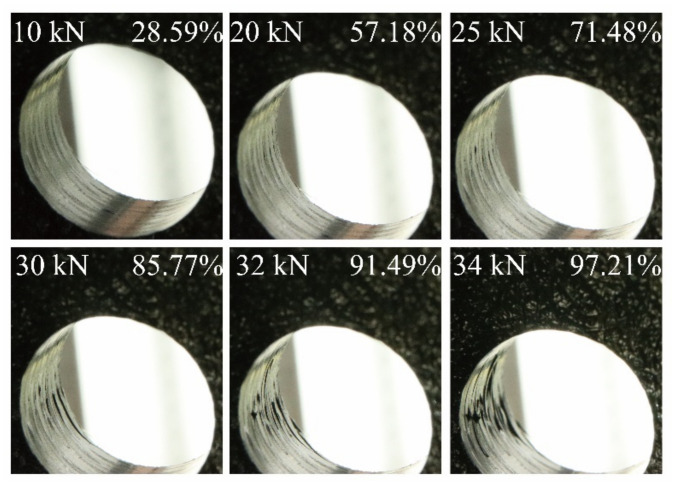
The experimental damage process at the open-hole region of the CY-4-01 specimen.

**Figure 16:**
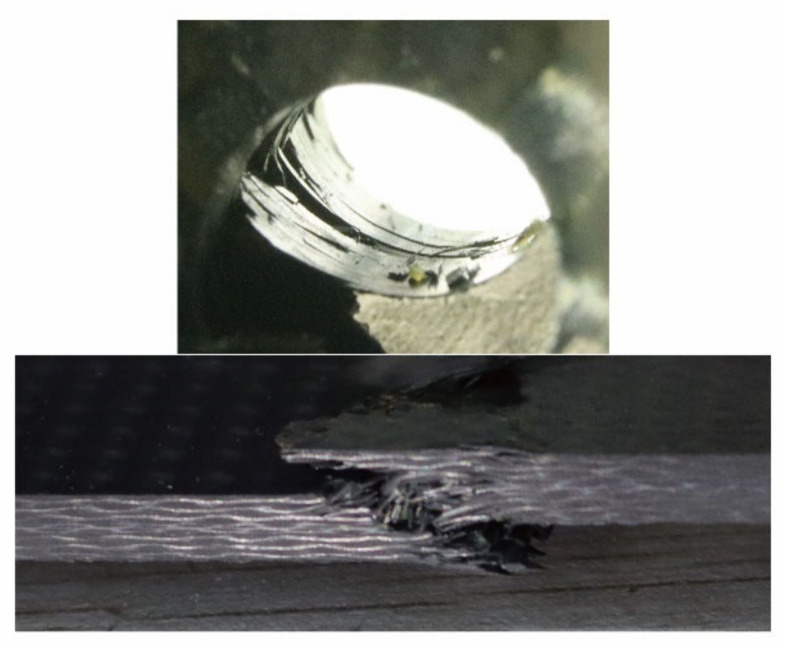
The ultimate failure of the CY-4-01 specimen.

The damage process at the open-hole region of the CY-4-01 specimen is presented in Figure 15. The damage statuses at the laminate’s open-hole region are presented, along with the corresponding compression load and its ratio between the experimental collapse load. At the initial loading stage, the compression load increases linearly with the shortening displacement and the status of the open-hole region remains smooth and regular with no damage occurs. As the compression load continues to grow, the damage of fiber initiates at the open-hole region with a slight amount of fiber extrusions detected at the compression load of 20 kN. In the subsequent loading process, the damage of the open-hole region intensifies and propagates along the radical direction of the open-hole. At the compression load of 30 kN, widely distributed damage including fiber extrusion and delamination can be clearly detected at the open-hole region. With the compression load further increases to 34 kN, the existing damage seriously aggravates. When the ultimate strength is reached, the laminate collapses with the loss of the carrying capacity and the fiber compressive failure can be detected along with the whole thickness of the specimen as shown in Figure 16.

## 5. Multi-Scale Numerical Simulation and Discussion

### 5.1. Finite Element Model

**Figure 17:**
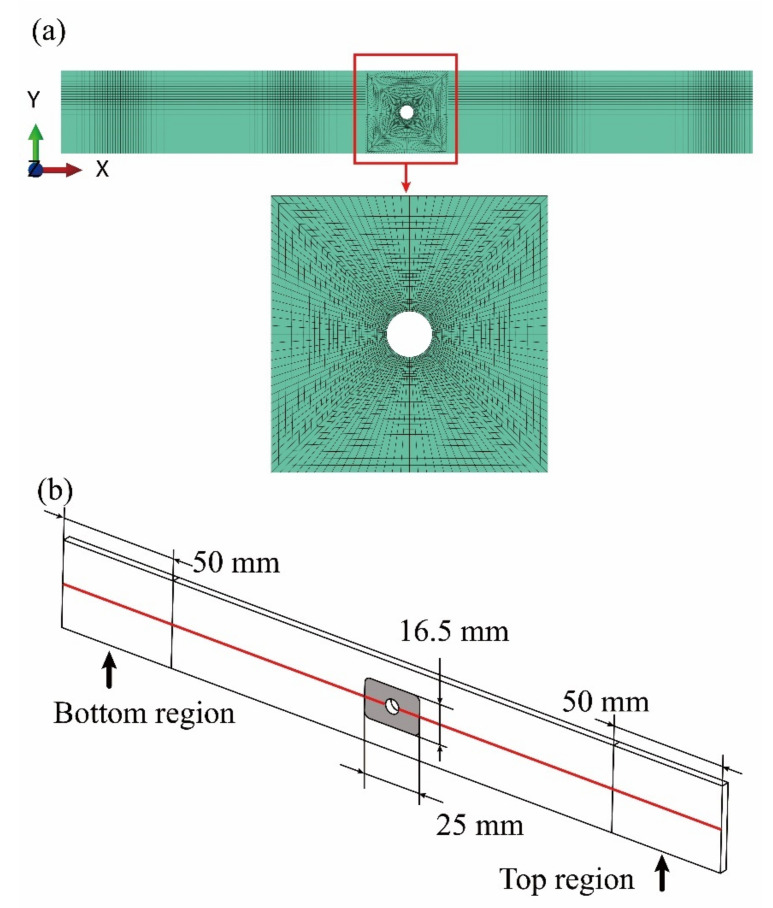
The macro-scale FE model of the T300/Cycom970 fabric open-hole laminates and the boundary conditions: (**a**) the macro-scale FE model of the open-hole laminates; (**b**) the boundary conditions.

With the strategy illustrated in Figure 12, a multi-scale finite element analysis has been implemented in modeling the compressive failure of the specimens of the open-hole fabric laminates. As shown in Figure 17a, the macro-scale FE model is discretized with the shell element of S4R in ensuring the calculation efficiency of the multi-scale analysis. With the consideration of calculation accuracy, 11,392 elements are finally used. The geometry parameters, the stacking sequence, and the material properties of the macro-scale FE model are consistent with the specimens, as introduced in Section 4 and listed in Table 5.

In order to simulate the clamping effects of the fixture and the axial compression boundary condition, the translation freedom in the z-direction of all the nodes is constrained except for the grey colored region around the centered open-hole. Besides, the translation freedom in the y-direction of the nodes in the middle red line is also constrained. The nodes in the bottom region of the FE model are coupled with a reference point and the translation freedom in the x-direction of the reference point is constrained. The displacement loading of compression in the x-direction is applied at the other reference point which is coupled with the nodes in the top region of the FE model. The boundary conditions of the macro-scale FE model are presented in Figure 17b.

The micro-scale RUC and the meso-scale RUC used for stress transformation are presented in Figure 3 and Figure 5b respectively and have been introduced above in Section 3.2.1 and Section 3.2.2. The modeling of damage initiation and damage evolution is conducted by using the ABAQUS user-defined field subordinate (USDFLD). The damage is only defined at the region of interest as amplified in Figure 17a with the aim to improve the calculation efficiency.

### 5.2. Numerical Results and Discussion

**Figure 18:**
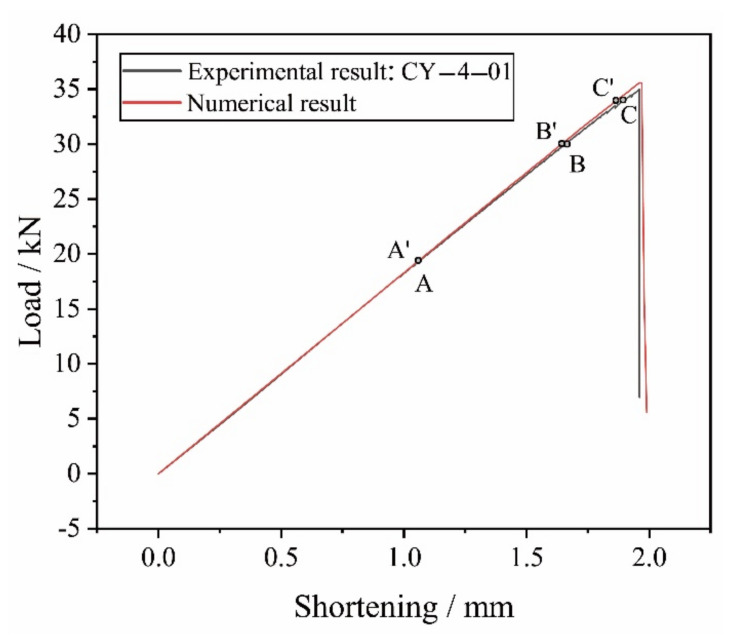
Comparison of the numerical and experimental results of load-shortening curves.

In Figure 18, the numerical load-shortening curve is compared with the experimental result of the CY-4-01 specimen. The numerical curve from the multi-scale finite element analysis is generally in good agreement with the experimental result and the numerical collapse load of 35.6 kN has a small deviation of comparing to the experimental average collapse load of 34.7 kN. From the beginning of the compression, the numerical result corresponds well with the experimental result at most parts of the curves, indicating the good capability of the FE model in modeling the linear bearing stage of the open-hole laminates. Since the interlaminar damage is not considered in the shell element based macro-scale FE model, the decrease of the laminate’s compression stiffness caused by the initiation and propagation of delamination cannot be reflected in the numerical result, making the slope of the numerical curve slightly higher than the slope of the experimental curve when the laminate approaches the final collapse.

On the experimental load-shortening curve of the CY-4-01 specimen, A, B and C are the points under the compression load of 20 kN, 30 kN, and 34 kN, respectively. A’, B’, and C’ are the points corresponding to A, B, and C, respectively on the numerical load-shortening curve. The predefined field variables (FV) are used in describing the intra-laminar damage and the numerical results indicate that the fiber compressive failure (described by FV2) is the dominant failure mode of the open-hole laminate.

**Figure 19:**
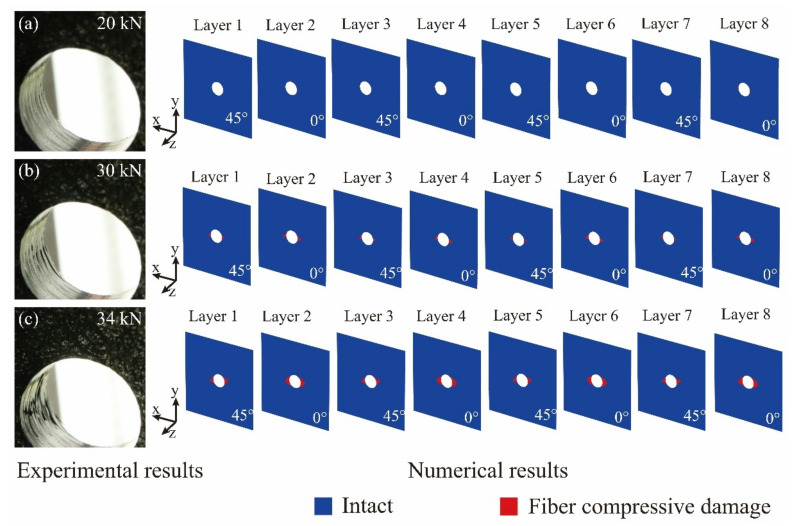
Comparison of the experimental and numerical damage statuses of the open-hole fabric laminate under different compression loads: (**a**) the compression load of 20 kN; (**b**) the compression load of 30 kN; (**c**) the compression load of 34 kN.

In Figure 19, the numerical fiber compressive damage statuses of the open-hole laminate at the points of A’, B’, and C’ in the numerical load-shortening curve are presented, together with the corresponding experimental damage statuses of the CY-4-01 specimen from the points of A, B, and C. Considering the symmetry of the laminates’ stacking sequence, the numerical results of the first 8 layers are presented. At the compression load of 20 kN in Figure 19a, the numerical results indicate that the fiber compressive damage initiates in the 0° plies within a small, limited region and the 45° plies remain intact. The calculated slight damage corresponds well with the recorded experimental phenomenon at the edge of the laminate’s open-hole. With the increase of the compression load, the fiber compressive damage propagates in the 0° plies and further extends to the 45° plies, as shown in Figure 19b. In Figure 19c, when the compression load reaches 34 kN and the laminated approaches the final collapse, the fiber compressive damage widely spreads along the radical direction in the 0° and 45° plies, and the serious experimental damage propagation is reflected by the numerical results.

**Figure 20:**
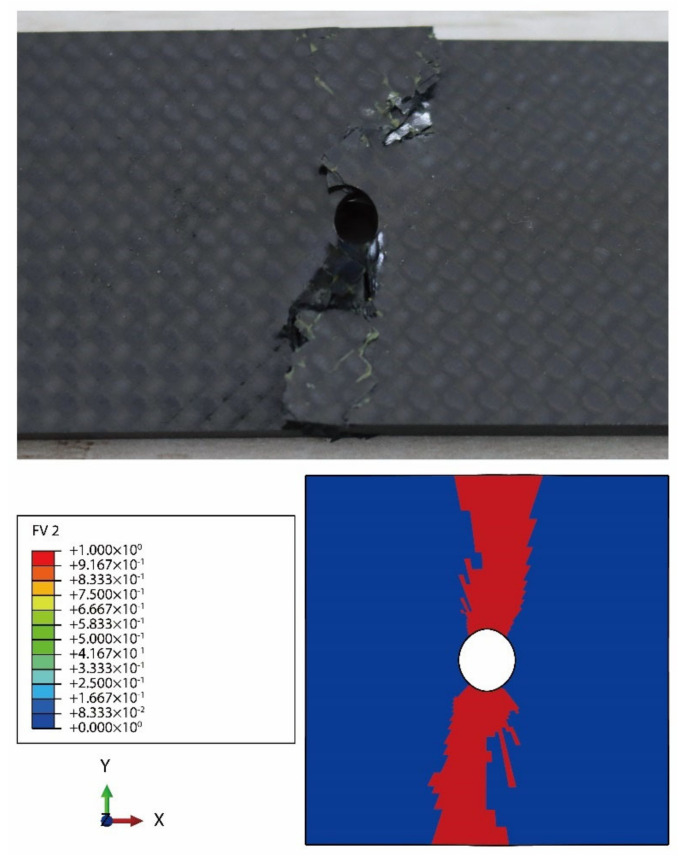
Comparison of the experimental and numerical failure statuses of the surface ply from the open-hole fabric laminate.

The failure status of the surface ply from the CY-4-01 specimen is compared with the numerical result in Figure 20, and it can be observed that the throughout fiber compressive damage along the transverse direction of the open-hole laminate is basically simulated by the numerical calculation.

As the damage process and the final collapse of the open-hole laminate are reasonably predicted by the numerical results of the multi-scale finite element analysis, the above comparisons demonstrate that the MMF3 criterion-based multi-scale strategy is effective in analyzing the failure behaviors of plain-woven fabric composite structures.

With a good engineering application prospect, this multi-scale strategy is planned to be used in the failure analysis of the plain-woven composite structures in our following research. As the factors of the randomness in the material and the irregular geometric properties of fiber and matrix constituents have unneglectable uncertainty effects on the mechanical performance of practical engineering composite structures, the multi-scale strategy is planned to work with the advanced technology of artificial neural networks [34] in its future engineering applications with the important uncertainty effects considered.

## 6. Conclusions

(1) With the aim to pursue greater accuracy and explore the damage mechanisms, a micromechanics-based multi-scale strategy is developed in this article in analyzing the failure behaviors of plain-woven fabric composites. Micro-scale stresses of the fiber and matrix constituents are achieved with the stress transformation methods and are imported into the MMF3 criterion in determining the failures at the micro-scale constituents’ level.
(2) The MMF3 criterion-based iteration methods have been developed in determining the necessary strengths used in the multi-scale finite element analysis for plain-woven fabric composites. The T300/Cycom970 composite is taken as the study object in this article. With the iteration methods and the assistance of the tested macro-scale strengths, the meso-scale strengths of the fiber tows and the micro-scale strengths of the constituents suitable for the T300/Cycom970 plain woven fabrics have been calculated, corrected, and validated.
(3) Compression experiments have been carried out on the T300/Cycom970 fabric open-hole laminates with the damage process recorded at the open-hole region. The experimental results are compared with the numerical results which are obtained from the MMF3 criterion-based multi-scale finite element analysis. With the good agreements between the experimental results and the numerical simulations in the aspects of the load-shortening curves, the damage process, and the final collapse, the effectiveness of the proposed multi-scale strategy has been demonstrated.

## Figures and Tables

**Table 1:** The MMF3 criterion in determining micro-scale failures.

Failure Type	Failure Criterion
Fiber failure	$\sigma_{11}\geq T_{f}\;\mathrm{or}\;\sigma_{11}\leq -C_{f}$
Matrix failure	$\left(\frac{1}{T_{m}}-\frac{1}{C_{m}}\right)\sigma_{I}+\frac{1}{T_{m}C_{m}}\sigma_{II}-\frac{1}{S_{m}{}^{2}}\sigma_{III}\geq 1$

**Table 2:** Mechanical properties of the fiber and matrix constituents in the T300/Cycom970 unidirectional laminates.

Constituent	Material Property	Value
Fiber (T300)	Longitudinal modulus, *E_f_*_11_ (GPa)	230.0
	Transverse modulus, *E_f_*_22_ = *E_f_*_33_ (GPa)	13.8
	In-plane shear modulus, *G_f_*_12_ = *G_f_*_13_ (GPa)	8.97
	Out-of-plane shear modulus, *G_f_*_23_ (GPa)	4.38
	$\text{In-plane}\;\mathrm{Poisson}’s\;\mathrm{ratio},\;\nu_{f12}=\nu_{f13}$	0.20
	$\text{Out-of-plane}\;\mathrm{Poisson}’s\;\mathrm{ratio},\;\nu_{f23}$	0.25
	$\mathrm{Longitudinal}\;\mathrm{coefficient}\;\mathrm{of}\;\mathrm{thermal}\;\mathrm{expansion},\;\alpha_{11}$ (10^−6^/K)	−0.54
	$\mathrm{Transverse}\;\mathrm{coefficient}\;\mathrm{of}\;\mathrm{thermal}\;\mathrm{expansion},\;\alpha_{22}=\alpha_{33}$ (10^−6^/K)	10.50
Matrix (Cycom970)	Elastic modulus, E*_m_* (GPa)	3.35
	$\mathrm{Elastic}\;\mathrm{Poisson}’s\;\mathrm{ratio},\;\nu_{m}$	0.35
	$\mathrm{Coefficient}\;\mathrm{of}\;\mathrm{thermal}\;\mathrm{expansion},\;\alpha_{m}$ (10^−6^/K)	58.00

**Table 3:** Tested macro-scale strengths of T300/Cycom970 unidirectional laminates.

Macro-Scale Strength	Value
Longitudinal tensile strength of unidirectional laminates, *X_T_* (MPa)	2050.5
Longitudinal compressive strength of unidirectional laminates, *X_C_* (MPa)	1345.3
Transverse tensile strength of unidirectional laminates, *Y_T_* (MPa)	81.1
Transverse compressive strength of unidirectional laminates, *Y_C_* (MPa)	221.6
In-plane shear strength of unidirectional laminates, *S*_12_ (MPa)	144.2

**Table 4:** Micro-scale strengths of the constituents for T300/Cycom970 unidirectional laminates.

Constituent	Micro-Scale Strength	Value
Fiber (T300)	Tensile strength, *T_f_* (MPa)	3598.6
	Compressive strength, *C_f_* (MPa)	2413.9
Matrix (Cycom970)	Tensile strength, *T_m_* (MPa)	149.3
	Compressive strength, *C_m_* (MPa)	340.3
	Shear strength, *S_m_* (MPa)	195.8

**Table 5:** Mechanical properties of the T300/Cycom970 plain-woven fabric composite laminates.

Property	Value
Longitudinal modulus, *E_F_*_11_ (GPa)	56.85
Transverse modulus, *E_F_*_22_ (GPa)	56.85
In-plane shear modulus, *G_F_*_12_ (GPa)	3.86
In-plane Poisson’s ratio, $\nu_{F12}$	0.042
Longitudinal tensile strength of fabric laminates, *X_T(F)_* (MPa)	717.0
Longitudinal compressive strength of fabric laminates, *X_C(F)_* (MPa)	597.0
Transverse tensile strength of fabric laminates, *Y_T(F)_* (MPa)	717.0
Transverse compressive strength of fabric laminates, *Y_C(F)_* (MPa)	597.0
In-plane shear strength of fabric laminates, *S*_12*(F)*_ (MPa)	128.0
Fiber volume fraction in tows, *V_f_*	0.78
Volume fraction of tows, *V_t_*	0.61
Total fiber volume fraction, *V_fT_*	0.48

**Table 6:** Meso-scale strengths of fiber tows in T300/Cycom970 fabrics calculated under the micro-scale strength invariant hypothesis.

Meso-Scale Strength	Value
Longitudinal tensile strength of fiber tows, *X_T(tow)_* (MPa)	2742.6
Longitudinal compressive strength of fiber tows, *X_C(tow)_* (MPa)	1821.8
Transverse tensile strength of fiber tows, *Y_T(tow)_* (MPa)	73.0
Transverse compressive strength of fiber tows, *Y_C(tow)_* (MPa)	162.5
In-plane shear strength of fiber tows, *S*_12*(tow)*_ (MPa)	134.7

**Table 7:** Geometric parameters of the meso-scale RUC from T300/Cycom970 fabrics.

Geometric Parameter	Value
Radius of the fiber tows’ lenticular cross-section, *r_tow_* (mm)	8.778
Width of the fiber tows’ lenticular cross-section, *W_tow_* (mm)	1.775
Thickness of the fiber tows’ lenticular cross-section, *T_tow_* (mm)	0.091
Radius of the fiber tows’ undulation path, *r_tow_* (mm)	8.832
Length of the meso-scale RUC, *L_RUC_* (mm)	3.754
Width of the meso-scale RUC, *W_RUC_* (mm)	3.754
Thickness of the meso-scale RUC, *T_RUC_* (mm)	0.216

**Table 8:** Mechanical properties of the fiber tows in the meso-scale RUC of the T300/Cycom970 plain-woven fabrics.

Property	Value
Longitudinal modulus, *E*_11*(tow)*_ (GPa)	174.67
Transverse modulus, *E*_22*(tow)*_ = *E*_33*(tow)*_ (GPa)	9.51
In-plane shear modulus, *G*_12*(tow)*_ = *G*_13*(tow)*_ (GPa)	8.52
Out-of-plane shear modulus, *G*_23*(tow)*_ (GPa)	3.39
$\text{In-plane}\;\mathrm{Poisson}’s\;\mathrm{ratio},\;\nu_{12\left(tow\right)}=\nu_{13\left(tow\right)}$	0.28
$\text{Out-of-plane}\;\mathrm{Poisson}’s\;\mathrm{ratio},\;\nu_{23\left(tow\right)}$	0.41
$\mathrm{Longitudinal}\;\mathrm{coefficient}\;\mathrm{of}\;\mathrm{thermal}\;\mathrm{expansion},\;\alpha_{11\left(tow\right)}$ (10^−6^/K)	−0.22
$\mathrm{Transverse}\;\mathrm{coefficient}\;\mathrm{of}\;\mathrm{thermal}\;\mathrm{expansion},\;\alpha_{22\left(tow\right)}=\alpha_{33\left(tow\right)}$ (10^−6^/K)	24.91

**Table 9:** Comparison of the meso-scale strengths of the fiber tows calculated from different methods.

	*X_T(tow)_*/MPa	*X_C(tow)_*/MPa	*S*_12*(tow)*_/MPa
Result calculated with the micro-scale RUC based on the micro strength invariant hypothesis	2742.6	1821.8	134.7
Result calculated with the meso-scale RUC based on the experimental macro strengths of the fabric laminates	2752.3	2292.5	139.1

**Table 10:** The meso-scale strengths of the fiber tows and the micro-scale strengths of the constituents suitable for the T300/Cycom970 plain-woven fabrics.

Meso-Scale Strength	Value	Micro-Scale Strength	Value
*X_T(tow)_*/MPa	2752.3	*T_f_*/MPa	3611.0
*X_C(tow)_*/MPa	2292.5	*C_f_*/MPa	3303.2
*Y_T(tow)_*/MPa	73.0	*T_m_*/MPa	149.5
*Y_C(tow)_*/MPa	162.5	*C_m_*/MPa	333.4
*S*_12*(tow)*_/MPa	139.1	*S_m_*/MPa	202.3

**Table 11:** Material property degradation scheme at the constituent level. Symbols on the left side of the equations with the superscript of * represent the degraded properties of the constituents and the degradation factors fit well with the experimental results.

Failure Type	Property Degradation
Fiber failure	*E_f_*_11_^*^ = 0.01 *E_f_*_11_, *E_f_*_22_^*^ = *E_f_*_22_, *E_f_*_33_^*^ = *E_f_*_33_ $\nu_{f12}$^*^ $=0.01\;\nu_{f12}$$,\;\nu_{f13}$^*^ $=0.01\;\nu_{f13}$$,\;\nu_{f23}$^*^ $=\nu_{f23}$ *G_f_*_12_^*^ = 0.01 *G_f_*_12_, *G_f_*_13_^*^ = 0.01 *G_f_*_13_, *G_f_*_23_^*^ = *G_f_*_23_,
Matrix failure	*E_m_* ^*^ $=0.05\;E_{m},\;\nu_{m}$ ^*^ $=\nu_{m}$

**Table 12:** Experimental results of the T300/Cycom970 fabric open-hole laminates.

Number of Specimen	Collapse Load (kN)	Average Value (kN)	Standard Deviation (kN)	Coefficient of Dispersion
CY-4-01	35.0	34.7	0.22	0.63%
CY-4-02	34.5			
CY-4-03	34.4			
CY-4-04	34.5			
CY-4-05	34.9			
CY-4-06	34.7			

## Data Availability

Data sharing is not applicable for this article.
